# Understanding and explaining the link between anthroposophy and vaccine hesitancy: a systematic review

**DOI:** 10.1186/s12889-023-17081-w

**Published:** 2023-11-13

**Authors:** Sibylle Herzig van Wees, Khadija Abunnaja, Sandra Mounier-Jack

**Affiliations:** 1 Department of Global Public Health, Karolinska Institute, Stockholm, Sweden; 2 Department of Global Health and Development, London School of Hygiene and Tropical, London, UK

**Keywords:** Vaccine confidence, Vaccine hesitancy, Anthroposophy

## Abstract

**Background:**

Due to low vaccination uptake and measles outbreaks across Europe, public health authorities have paid increasing attention to anthroposophic communities. Public media outlets have further described these communities as vaccine refusers or “anti-vaxxers”. The aim of this review was to understand the scope of the problem and explore assumptions about vaccination beliefs in anthroposophic communities. For the purpose of this review, we define anthroposophic communities as people following some/certain views more or less loosely connected to the philosophies of anthroposophy. The systematic review addresses three research questions and (1) collates evidence documenting outbreaks linked to anthroposophic communities, (2) literature on vaccination coverage in anthroposophic communities, and (3) lastly describes literature that summarizes theories and factors influencing vaccine decision-making in anthroposophic communities.

**Methods:**

This is a systematic review using the following databases: Medline, Web of Science, Psycinfo, and CINAHL. Double-blinded article screening was conducted by two researchers. Data was summarized to address the research questions. For the qualitative research question the data was analysed using thematic analysis with the assistance of Nvivo12.0.

**Results:**

There were 12 articles documenting 18 measles outbreaks linked to anthroposophic communities between the years 2000 and 2012. Seven articles describe lower vaccination uptake in anthroposophic communities than in other communities, although one article describes that vaccination coverage in low-income communities with a migrant background was lower than in the anthroposophic community they studied. We found eight articles examining factors and theories influencing vaccine decision making in anthroposophic communities. The qualitative analysis revealed four common themes. Firstly, there was a very broad spectrum of vaccine beliefs among the anthroposophic communities. Secondly, there was a consistent narrative about problems or concerns with vaccines, including toxicity and lack of trust in the system. Thirdly, there was a strong notion of the importance of making individual and well-informed choices as opposed to simply following the masses. Lastly, making vaccine choices different from public health guidelines was highly stigmatized by those outside of the anthroposophic community but also those within the community.

**Conclusion:**

Continuing to further knowledge of vaccine beliefs in anthroposophic communities is particularly important in view of increasing measles rates and potential sudden reliance on vaccines for emerging diseases. However, popular assumptions about vaccine beliefs in anthroposophic communities are challenged by the data presented in this systematic review.

**Supplementary Information:**

The online version contains supplementary material available at 10.1186/s12889-023-17081-w.

## Background

Vaccines save lives [1]. Vaccine hesitancy has been considered one of the top 10 public health threats of our time [2]. For the purpose of this study, we define vaccine hesitancy as the delay or refusal of vaccines despite their availability [3]. In recent years, public health agencies and researchers have paid increasing attention to vaccination beliefs of anthroposophical communities. For example, the Public Health Agency in Sweden has identified an anthroposophic community outside of Stockholm as a group of concern regarding low vaccination uptake [4]. The interest in this group’s vaccination beliefs and behaviours is mainly due to a growing number of measles outbreaks in anthroposophic communities across Europe [5]. In this study, we provide an overview of existing published evidence that examines the relationship between anthroposophy and vaccine beliefs. We focus on individuals and groups who follow an anthroposophical lifestyle or are inspired by anthroposophy. This includes communities that attend Waldorf/Steiner schools. It is important to note that the scope of adherence to principles on anthroposophy varies significantly between individuals and we recognize the diversity within this community.

Anthroposophy is a spiritualist movement that was established by scientist and philosopher Rudolf Steiner born in 1861 in Austria [6]. Anthroposophy literally implies wisdom about man, and stipulates that through meditation and concentration, individuals can utilize the physical world to connect with the spiritual world [6–10]. Steiner believed that man’s (sic) own thinking was the path to spiritual and inner observations [9, 10]. The spiritualist movement began in Germany in the early twentieth century and ideas of anthroposophy have been applied to many areas of life such as education, art, architecture, and healthcare [6] and led to the creation of now well-established Waldorf schools and anthroposophic medicine worldwide [7]. For example, there are over 1000 Waldorf schools (also referred to as Steiner schools) in around 60 countries around the world [2].

Anthroposophic medicine was founded in the early 1920s by Rudolf Steiner and Ita Wegman [6]. Drawing on anthroposophic philosophy it incorporates a holistic approach to the understanding of illness and approaches to healing [6, 7]. Anthroposophic medicine addresses a broad spectrum of health issues (family medicine, chronic disease, paediatric disease and palliative care) and is offered in combination with mainstream medicine or in anthroposophic medical practices [6]. It offers medicines derived from herbs, minerals, animals, eurythmy and art therapy, massage, and, counselling and psychotherapy [11]. Anthroposophic medicine can be studied at accredited schools by medical doctors, movement and mental health therapists and nurses [7].

Anthroposophic medicine is practiced in 78 countries worldwide, predominantly in Central Europe. There are circa 24 anthroposophic medical institutions – these include hospitals, departments in hospitals, rehabilitation centres, and other inpatient healthcare centres in six countries (Germany, Switzerland, Sweden, Italy, The Netherlands, and The United States) [7]. Moreover, there are around 180 anthroposophic outpatient clinics globally where anthroposophic physicians work in collaboration with biomedical approaches to health care. In addition, anthroposophic physicians work in their own practices or in collaboration with other complementary health care providers [7]. In Germany, Latvia, and Switzerland, anthroposophic medicine is considered a distinct and specialized therapy. In Germany, it is overseen by its own committee at the Federal Institute for drugs and medical devices. Anthroposophic medicine is popular and in some instances revealed higher patient satisfaction compared to conventional health care [7].

### Anthroposophic medicine and vaccination

The 2019 official statement of the international centre of anthroposophic medicine, the Medical Section of the Goetheanum, and the International Federation of Anthroposophic Medical Associations (IVAA) clearly states that they do not support the anti-vaccine movement. Rudolf Steiner did not oppose vaccines, however, vaccination and anthroposophic medicine constitutes a somewhat contentious point [11]. This is partly because Rudolf Steiner argued that childhood illnesses are important for growth and development of a child, leading some to question the necessity of vaccines [6, 11]. In the past decade, concerns have been raised by the scientific community on the role of the anthroposophic movement in measles outbreaks [12–14]. Consequently, in some countries such as Sweden and Germany, anthroposophic communities have been labelled as a community that refuses vaccines, particularly by popular media and during the COVID-19 pandemic [15–17]. Despite this attention from public media and science, there is no comprehensive review on the scope of the problem, in terms of number of outbreaks and vaccination coverage in anthroposophic communities. Whilst there are a number of qualitative studies that elucidate the factors influencing vaccine decision making in anthroposophic communities, there is no systematic and comparative review of this evidence.

Therefore, this review aims to understand the scope of the problem and explore popular assumptions about vaccine beliefs in anthroposophic communities. To achieve this, this systematic review summarizes the existing literature that investigates the relationship between anthroposophy and vaccination beliefs.

## Methods

### Design

This is a systematic review, including both quantitative and qualitative studies. The review is based on current best practices utilising the Joanna Briggs Institute systematic review framework [18, 19].

We used the population/concept/context (PCC) framework to guide the development of our research questions [18]. The population being anthroposophic communities; the concept vaccine hesitancy or vaccine confidence/trust; context including a global setting. This framework as well as the literature review culminated in three research questions:

1. What are the documented outbreaks associated with low vaccination coverage in anthroposophic communities?
2. What is the evidence for vaccination rates in anthroposophic communities?
3. What is the evidence that describes factors and theories for low vaccination uptake in anthroposophic communities?

### Protocol and registration

No review protocol exists, and the systematic review has not been registered.

### Search strategy

A systematic literature search was performed by two researchers in the following databases: Medline, Web of Science, Psycinfo, and CINAHL. The last search was conducted 2022–09-05. The search strategy was developed in Medline (Ovid) in collaboration with librarians at the Karolinska Institutet University Library. For each search concept Medical Subject Headings (MeSH-terms) and free text terms were identified (see appendix). No language restriction was applied. Databases were searched from inception. The strategies were peer reviewed by another librarian prior to execution. De-duplication was done using the method described by Bramer et al. [20]. One final, extra step was added to compare DOIs to ensure no duplication. The full search strategies for all databases are available in the Appendix.

### Study selection and inclusion and exclusion criteria

Independent study selection was completed by two reviewers (SHvW and KA). Inclusion criteria for the first round of screening (title and abstract) were all articles that discussed anthroposophy and vaccination (this was conducted by KA and SHvW). Articles in Swedish and German were only reviewed by SHvW due to language restrictions. Inclusion criteria for the second and more in-depth round of screening – conducted double-blinded by KA and SHvW were all papers relevant to the three research questions, including both quantitative and qualitative studies. Exclusion criteria were, not peer-reviewed papers, opinion pieces, systematic reviews, nor papers that were not relevant to the research question (for example there were a number of articles that examined the relationship between anthroposophy, vaccination, and allergy).

### Quality assessment

A quality assessment of the selected papers was conducted double-blinded by two researchers (KA and SHvW). For the qualitative studies, we used the JBI Critical Appraisal Tool for Qualitative Research [19]. This is a quality control checklist. For the quantitative studies, we applied the Effective Public Health Practice Project quality evaluation tool to assess the quality of all quantitative publications that were included as references in this work [21]. Each article received a final rating at the conclusion with one of the following scores: 1 (Strong), 2 (Moderate), or 3 (Weak), based on an assessment of study design, methods used, sampling, and bias [19, 20]. We then calculated the average of the results (from reviewers KA and SHvW), which represents the overall evaluation of the quality of all quantitative papers. The articles included received an overall score of 1.9. This indicates that there was a moderate quality of research papers presented in this review.

### Analysis

To address research questions one and two, the data was summarized in Tables 1, 2 and 3. To address research question three, the qualitative research data of the articles included in this review (Table 4) were analysed using Braun and Clarke’s thematic analysis [22] with the support of Nvivo12. The data (Results from articles) were imported into Nvivo12, coded and categories from the grouping of codes were created (double blinded) by KA and SHvW. The creation of themes was discussed between KA and SHvW. The coding tree is presented in Table 5.

**Table 1:** Measles outbreaks linked to anthroposophic communities

Place	Year	Cases in anthroposophic	Outbreak total n	Origin	Catch-up strategy
Järna, Sweden [23]	2012	16	N/A	Unclear^a^	N/A
Falunders, Belgium [24]	2011	41	65	Pre-school, spread to Waldorf school	Vaccination campaigns and discussion with an anthroposophic clinic Isolation of cases was successful. Spread could be halted
Offenburg, Germany [25]	2011	34	34	Waldorf school	Closure of school
Freiburg [25]	2011	5	5	Waldorf school	If children were unvaccinated they could not attend school until epidemic was over
Berlin [26]	2011	20	73	From community to Waldorf school	School exclusion of unvaccinated children was swiftly implemented
Berlin, Germany [25]	2010	62	62	Waldorf school	N/A
Essen, Germany [25]	2010	30	71	Waldorf School	N/A
Styria, Austria [27]	2009	12	25	From general population to Waldorf school	Prompt two-week closure of the anthroposophic school and the prompt isolation of cases at home for the period of communicability
Salzburg, Austria [5,25, 28]	2008	123	394	Waldorf school student from Switzerland	School closure, isolation, offering MMR vaccination free of charge to the population younger than 15 years
Germany [28]	2008	53	394	Waldorf school student from Switzerland (across Waldorf schools)	School closure, isolation, offering MMR vaccination free of charge to the population younger than 15 years
Norway [28]	2008	4	394	Waldorf school student from Switzerland	School closure, isolation, offering MMR vaccination free of charge to the population younger than 15 years
Freiburg, Germany [25]	2008	60	60	Waldorf school	N/A
Netherlands [29]	2008	36	36	Waldorf school	Vaccination catch up. Very low uptake n = 10; information letter to parents
Gerresheim [25]	2007	4	4	Waldorf school	Vaccination campaign on campus was offered but school declined this
Switzerland [5]	2006–2007	N/A	N/A	The outbreak involved schools, an anthroposophic boarding school and daycare centers	N/A
Cobug [12, 25]	2003	N/A	1191	Waldorf school	N/A
UK [12]	2000	N/A	293	A child visiting an anthroposophic community	
Netherlands [12]	1999/2000	100	3292	N/A	N/A

^a^Whilst the article states that the outbreak happened within the community, there is insufficient detail from the literature to conclude the starting point

## Results

**Fig. 1:**
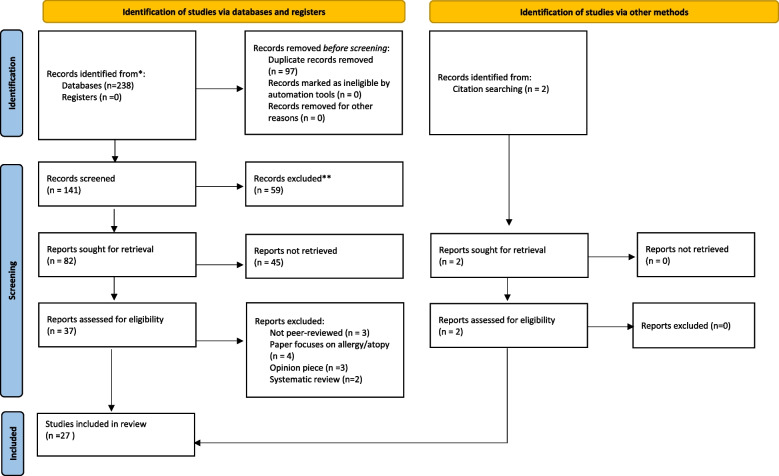
Prisma flowchart

The search revealed 27 papers (see Fig. 1). Twelve papers describe 18 outbreaks associated with anthroposophic communities. Seven papers describe vaccination coverage/personal belief exception rates associated with anthroposophic beliefs. Eight papers describe factors influencing vaccine decision-making among anthroposophic communities and anthroposophic providers.

### Outbreaks in anthroposophic communities

Table 1 describes 18 measles outbreaks that occurred between 1997 and 2011 in European countries, which were described in 12 studies (Table 2 summarizes the papers).^1^ Table 1 describes the location, the outbreak year, the number of cases, the source of the outbreak, and any catch-up strategies (where described). The studies show that eight out of 18 measles outbreaks started at Waldorf schools throughout Germany, Switzerland, Austria, Netherlands, and the UK [8, 17, 20, 22]. Although data from community reporting is limited, in the articles described, the measles cases at Waldorf schools are predominantly higher than in mainstream private or state schools across the five countries. Offering measles vaccination catch-ups by public health authorities (which is an effective way to manage a measles outbreak) was described in several articles but was largely refused by both parents and Waldorf schools. The most effective outbreak control strategy was the immediate closure of the Waldorf school and strict rules regarding entry to the school upon reopening.

**Table 2:** Summary of 12 articles describing outbreaks associated with anthroposophic medicine/Waldorf schools

Paper	Country	Year	Disease and Cases	Study aim	Population
Braeye, T., Sabbe, M., Hutse, V., Flipse, W., Godderis, L. and Top, G., 2013. Obstacles in measles elimination: an in-depth description of a measles outbreak in Ghent, Belgium, spring 2011. *Archives of Public Health*, *71(1)*, pp.1–7	Belgium	2011	65 measles cases	This report describes a measles outbreak and evaluates control measures and interventions	The outbreak was in Flanders. It started in a day care center, infecting children too young to be vaccinated, spread to anthroposophic schools with a low vaccination coverage
Ernst, E., 2011. Anthroposophic medicine causes measles outbreaks. *Deutsche Medizinische Wochenschrift (1946)*, *136(44)*, pp.2271–2272	Germany	2011	Describes 7 measles outbreaks in Europe	Summarizes measles outbreaks	Waldorf school
Ernst, E., 2011. Anthroposophy: a risk factor for noncompliance with measles immunization. *The Pediatric infectious disease journal*, *30*(3), pp.187–189	In several European countries: Germany, Austria, Netherlands, and Great Briain	2011	Describes 5 measles outbreaks in Europe	Review of outbreaks	Waldorf schools
Lassen, S.G., Schuster, M., Stemmler, M., Steinmüller, A., Matysiak-Klose, D., Mankertz, A., Santibanez, S., Wichmann, O. and Falkenhorst, G., 2014. Measles outbreak spreading from the community to an anthroposophic school, Berlin, 2011. *Epidemiology & Infection*, *142*(4), pp.789–796	Berlin	2011	73 measles cases	Measles outbreak investigation in Berlin 2011:	Berlin community and among students of an anthroposophic school
Muscat, M., 2011. Who gets measles in Europe?. *The Journal of Infectious Diseases*, *204*(suppl_1), pp.S353-S365	Several European countries	2011	Describes 4 measles outbreaks linked to anthropsophic communities		Individuals susceptible to measles
Roggendorf, H., Mankertz, A., Kundt, R. and Roggendorf, M., 2010. Spotlight on measles 2010: Measles outbreak in a mainly unvaccinated community in Essen, Germany, March–June 2010. *Eurosurveillance*, *15*(26), p.19605	Germany	2010	71 measles cases	Describes measles outbreak in 2010 in Germany and lists public health interventions used to stop this outbreak	Unvaccinated community in Essen Cases identified in members of the Waldorf school or kindergarten, siblings of those members and visiting doctors who do not recommend vaccination
Kasper, S., Holzmann, H., Aberle, S.W., Wassermann-Neuhold, M., Gschiel, H., Feenstra, O., Allerberger, F. and Schmid, D., 2009. Measles outbreak in Styria, Austria, march–may 2009. *Eurosurveillance*, *14*(40), p.19347	Austria	2009	37 cases in 2009, 397 cases in 2008	Outbreak investigation: to describe the measles outbreak by person, place and time and to identify the proportion of cases who were vaccinated	General population and the anthroposophic community in the Austrian province of Styria
Schmid, D., Holzmann, H., Abele, S., Kasper, S., König, S., Meusburger, S., Hrabcik, H., Luckner-Hornischer, A., Bechter, E., DeMartin, A. and Stirling, J., 2008. An ongoing multi-state outbreak of measles linked to non-immune anthroposophic communities in Austria, Germany, and Norway, March–April 2008. *Eurosurveillance*, *13*(16), p.18838	Austria, Germany, Norway	2008	202 measles cases in Austria, 53 in Germany, and four in Norway, *total number of measles cases 259*	Outbreak investigation	Non-immune anthroposophic communities in Austria, Germany, and Norway
Schmid, D., Holzmann, H., Schwarz, K., Kasper, S., Kuo, H.W., Aberle, S.W., Redlberger-Fritz, M., Hautmann, W., Santibanez, S., Mankertz, A. and König, C., 2010. Measles outbreak linked to a minority group in Austria, 2008. *Epidemiology & Infection*, *138*(3), pp.415–425	Austria	2008	A total of 394 (cases fulfilled the outbreak case definition} including 168 (affiliated to the anthroposophic community)	Described measles outbreak	Anthroposophic school in Salzburg city (Austria)
Wadl, M., Siedler, A., Krämer, W., Haindl, M.E., Gebrande, S., Krenn-Lanzl, I., Mankertz, A. and Hautmann, W., 2011. Measles transmission from an anthroposophic community to the general population, Germany 2008. *BMC Public Health*, *11*(1), pp.1–8	Germany	2008	(217 Bavarian cases identified) (28 cases were attendees of the anthroposophic school in Austria)	Measles Outbreak investigation and its transmission to the general population which will guide the future public health action	Two neighbouring Bavarian counties with students attended an Austrian anthroposophic school
Hanratty, B., Holt, T., Duffell, E., Patterson, W., Ramsay, M., White, J.M., Jin, L. and Litton, P., 2000. UK measles outbreak in non-immune anthroposophic communities: the implications for the elimination of measles from Europe. *Epidemiology & Infection*, *125*(2), pp.377–383	UK	2000	293 measles cases	Describes the epidemiology of this UK measles outbreak	Non-immune anthroposophic communities, and unvaccinated groups
Ströhle, A., Eggenberger, K., Steiner, C.A., Matter, L. and Germann, D., 1997. Mumps epidemic in vaccinated children in West Switzerland. *Schweizerische Medizinische Wochenschrift*, *127*(26), pp.1124–1133	Switzerland	1997	Exact number of cases not present but describes Mumps outbreaks throughout Switzerland	Describing the epidemic of Mumps in Switzerland and the main interacting factors that led to the epidemic	Vaccinated and unvaccinated children in Switzerland

Table 2 summarizes 12 articles that describe outbreaks in Europe linked to anthroposophic communities. Eleven articles describe the 18 measles outbreaks identified, and some of the outbreaks are mentioned in several papers. One article describes a mumps outbreak in Switzerland in the 1990s.

### Vaccination coverage in anthroposophic communities

Table 3 summarizes six articles that describe vaccine coverage in anthroposophic communities, and one article describes the personal belief exception (PBE) rate at Waldorf school in the USA. The papers focus predominantly on diphtheria, pertussis, tetanus and poliomyelitis (DPTP), and mumps, measles and rubella (MMR) vaccines. Two studies studying the vaccination coverage at Waldorf pre-schools/schools, demonstrate overall low immunization coverage at those schools [30, 31]. One article focusing on PBE rates demonstrates a proportionally high rate at Waldorf schools in California [32]. Three studies from the Netherlands measure vaccination coverage in general and focus specifically on whether there are special groups that show specifically low coverage [33–35]. In these studies, anthroposophic communities are identified as showing low coverage [14–16]. However, one study highlights that anthroposophic communities are not as significant in terms of low coverage as low-income groups [33]. One paper describes rates of vaccination refusal in Switzerland [36]. It highlights that complementary alternative medicine (CAM) users, including people who draw on anthroposophic medicine, are more likely to refuse vaccination. However, the paper also shows that this group was more likely to vaccinate against tick-borne diseases and encephalitis than the general population [36].

**Table 3:** Summary of seven articles describing vaccination coverage and uptake in anthroposophic communities

Paper	Country	Year	% vaccination coverage /PBE exemption rates	Study aim & population	Key message
Pfaff, G., Leher, A., Fechler, A. and Ouédraogo, N., 2017. Immunization coverage among children in Waldorf kindergartens, South West Germany 2015-2016Gunter Pfaff. *European Journal of Public Health*, *27*(suppl_3)	Germany	2017	We analyzed school entry health examination records of 91.653 children seen in 2014/2015 by either PHS staff (PHS, *n* = 90 653) or by physicians of Waldorf kindergartens (Waldorf, *n* = 1 247) Absence of immunization coverage varied for poliomyelitis (PHS 2,5%/Waldorf 20,2%), diphtheria (1,8%/12,5%), tetanus (1,0%/4,6%), pertussis (2,6%/23,9%), haemophilus influenzae b (3,7%/29,4%), varicella (16,4%/60,1%), hepatitis B (9,8%/48,6%), pneumococcal vaccine (13,1%/51,6%), meningococcus C (13,6%/56,4%) and tick borne encephalitis (65,2%/87,0%). Coverage with measles containing vaccine (MCV) varied for no dose (4,9%/34,0%), first dose (95,1%/66,0%) and two doses MCV (89,2%/55,1%) MCV coverage among Waldorf children had a calculable impact on state wide MCV coverage (94,7%)	Estimating the immunization coverage among children aged four to five years for Waldorf kindergartens	Low immunization coverage among children in Waldorf kindergartens continues to be a risk indicator for outbreaks of vaccine preventable diseases
Brennan, J.M., Bednarczyk, R.A., Richards, J.L., Allen, K.E., Warraich, G.J. and Omer, S.B., 2017. Trends in personal belief exemption rates among alternative private schools: Waldorf, Montessori, and holistic kindergartens in California, 2000–2014. *American journal of public health*, *107*(1), pp.108–112	USA	2017	Alternative schools had an average Personal Belief Exception (PBE) rate of 8.7%, compared with 2.1% among public schools. **Waldorf schools had the highest average PBE rate of 45.1%,** which was 19 times higher than in public schools (incidence rate ratio = 19.1; 95% confidence interval = 16.4, 22.2) Montessori and holistic schools had the highest average annual increases in PBE rates, slightly higher than Waldorf schools (Montessori: 8.8%; holistic: 7.1%; Waldorf: 3.6%)	To evaluate trends in rates of personal belief exemptions (PBEs) to immunization requirements at private kindergartens in California that practice alternative educational methods	Waldorf schools had exceptionally high average PBE rates
Klomp, J.H., van Lier, A. and Ruijs, W.L., 2015. Vaccination coverage for measles, mumps and rubella in anthroposophical schools in Gelderland, The Netherlands. *The European Journal of Public Health*, *25*(3), pp.501–505	The Netherlands	2015	The mean self-reported MMR vaccination coverage in 2012: 83% (95% CI: 79–86%), individual schools (range 45–100%) in anthroposophical schools in 2014 was 78% (95%CI: 77–80%) and varied less (range 59–88%)	Assess the MMR vaccination coverage in 11 anthroposophical schools in Gelderland, The Netherlands	Social clustering of unvaccinated children in anthroposophical schools remains a public health challenge
Van der Wal, M.F., Diepenmaat, A.C.M., Pel, J.M. and Hirasing, R.A., 2005. Vaccination rates in a multicultural population. *Archives of disease in childhood*, *90*(1), pp.36–40	The Netherlands	2005	The vaccination rates for children between age 5–12 living in Amsterdam in 2003 in the various districts ranged between 79.0% and 99.4% for DPTP and 81.9% and 98.4% for MMR High proportion (28%) of children attending anthroposophic schools who are not MMR vaccinated	To establish whether there are social or cultural groups of children in Amsterdam with relatively low vaccination coverage for (DPTP), (MMR)	Children who attended anthroposophical schools were found to be considerably less frequently fully immunised than those at other types of schools
Van der Wal, M.F., Diepenmaat, A.C., Pauw-Plomp, H. and van Weert-Waltman, M.L., 2001. High vaccination rates among children of Amsterdam. *Nederlands Tijdschrift Voor Geneeskunde*, *145*(3), pp.131–135	The Netherlands	2001	Children who visited anthroposophical schools were considerably less immunized compared with children visiting other schools: for DPTP and MMR 81.0 and 59.9% respectively versus 94.4, 95.3% for children attending general municipal schools	To examine if in Amsterdam there are social or cultural groups of children with a relatively low vaccination coverage for diphtheria, pertussis, tetanus and poliomyelitis (DPTP) and mumps, measles and rubella (MMR)	Children who visited anthroposophical schools were considerably less immunized compared with children visiting other schools
Mollema L, Wijers N, Hahné SJ, van der Klis FR, Boshuizen HC, de Melker HE. Participation in and attitude towards the national immunization program in the Netherlands: data from population-based questionnaires. BMC public health. 2012 Dec;12(1):1–3	The Netherlands	2013	Ethnicity, religion, income, educational level and anthroposophic beliefs were important determinants of nonparticipation in the NIP	The aim of this study was to determine which factors were associated with nonparticipation in the NIP and which ones were associated with parents' intention to accept remaining vaccinations Quantitative (*n* = 458)	Groups with a lower income or educational level or of non-Western descent participated less in the NIP than those with a high income or educational level or indigenous Dutch and have been less well identified previously. Particular attention ought to be given to these groups as they contribute in large measure to the rate of nonparticipation in the NIP, i.e., to a greater extent than well-known vaccine refusers such as specific religious groups and anthroposophics
Zuzak TJ, Zuzak-Siegrist I, Rist L, Staubli G, Simões-Wüst AP. Attitudes towards vaccination: users of complementary and alternative medicine versus non-users. Swiss medical weekly. 2008;138(47–48):713–8	Switzerland	2008	Refusal of basic vaccination was significantly more frequent among CAM (Complementary and Alternative Medicine)-users than among non-users (18.2% versus 3.5%, *p* (18.2% versus 3.5%, p < 0.001). The highest frequencies of refusal were reported by patients who consulted physicians practicing herbal medicine, anthroposophical medicine or homeopathy. Users and non-users of CAM however, showed comparable rates of immunisation in the case of the vaccinations against invasive meningococcal, pneumococcal disease and flu. Surprisingly, the rate for vaccination against tick-borne encephalitis was higher in the CAM-users group than among the non-users (21.2% versus 15.4%, p	To understand parental decisions behind basic vaccination refusal (*n* = 1158)	Refusal of basic vaccination was significantly more frequent among CAM (Complementary and Alternative Medicine)-users than among non-users. CAM users reported high tick-borne encephalitis vaccine choices

### Factors and theories influencing vaccine decision making in anthroposophic communities

The systematic search revealed eight articles examining factors and theories influencing vaccine decision-making in anthroposophic communities (see Table 4). Five articles focused on parents of children attending Waldorf schools or who considered themselves part of an anthroposophic community. Three articles focused on the perspectives of anthroposophic healthcare providers [37, 38], although two of those articles mixed and compared views with other alternative/complementary providers or allopathic health providers. Of the eight articles, two were quantitative [33, 39] and did not provide an in-depth discussion. The qualitative findings from six articles [23, 38, 40–42] were summarized in-depth and revealed four themes (see Table 5).

**Table 4:** Summary of eight studies addressing factors associated with vaccination decision making in anthroposophic communities

Mollema L, Wijers N, Hahné SJ, van der Klis FR, Boshuizen HC, de Melker HE. Participation in and attitude towards the national immunization program in the Netherlands: data from population-based questionnaires. BMC public health. 2012 Dec;12(1):1–3	The Netherlands	2013	The aim of this study was to measure vaccination coverage for MMR vaccines among children attending anthroposophical schools and gain more insight on attitudes towards childhood vaccination of parents with children attending anthroposophical schools	Quantitative (*n* = 458)	Parents of children attending anthroposophic schools
Byström E, Lindstrand A, Likhite N, Butler R, Emmelin M. Parental attitudes and decision-making regarding MMR vaccination in an anthroposophic community in Sweden–a qualitative study. Vaccine. 2014 Nov 28;32(50):6752–7	Sweden	2014	To explore facilitators and barriers to MMR vaccination among parents living in anthroposophic communities in Sweden	Qualitative: 19 semi-structured interviews	Parents in an anthroposophic community
Duffell E. Attitudes of parents towards measles and immunisation after a measles outbreak in an anthroposophical community. Journal of Epidemiology & Community Health. 2001 Sep 1;55(9):685–6	UK	2001	To explore attitudes of parents towards measles and immunisation after a measles outbreak in an anthroposophical community	Quantitative: Survey *n* = 126	Parents in an anthroposophical community in Gloucestershire
Harmsen IA, Ruiter RA, Paulussen TG, Mollema L, Kok G, de Melker HE. Factors that influence vaccination decision-making by parents who visit an anthroposophical child welfare center: a focus group study. Advances in preventive medicine. 2012 Jan 1;2012	The Netherlands	2012	To explore the beliefs underlying their childhood vaccination decision-making	Qualitative, 3 Focus Group Discussion (*n* = 16)	Parents who Visit an Anthroposophical Child Welfare Center
Mittring-Junghans N, Holmberg C, Witt CM, Teut M. Thoughts, beliefs and concepts concerning infectious childhood diseases of physicians practicing homeopathic, anthroposophic and conventional medicine–a qualitative study. BMC Complementary Medicine and Therapies. 2021 Dec;21(1):1–9	Germany	2021	To investigate the concepts and beliefs toward infectious childhood diseases among physicians practicing conventional, homeopathy and anthroposophic medicine	Qualitative, In-depth interviews (6 homeopathic, 6 anthroposophic and 6 conventional)	Health providers
Mollema L, Staal JM, van Steenbergen JE, Paulussen TG, de Melker HE. An exploratory qualitative assessment of factors influencing childhood vaccine providers' intention to recommend immunization in the Netherlands. BMC Public Health. 2012 Dec;12(1):1–0	Netherlands	2012	To examine factors related to providers’ intentions to recommend vaccinations to parents of young children	Qualitative, 4 Focus group discussions, only ***1 dicussion with providers at an anthroposophic welfare centre***	Health providers
Sobo, E.J., 2015. Social cultivation of vaccine refusal and delay among Waldorf (Steiner) school parents. *Medical anthropology quarterly*, *29*(3), pp.381–399	USA	2015	To help explain this PBE rate and inform interventions	Qualitative, 2 focus groups and conducted six formative and 18 cognitive interviews, Survey of vaccine preferences (*n* = 36)	Parents at a Waldorf School
Deml MJ, Notter J, Kliem P, Buhl A, Huber BM, Pfeiffer C, Burton-Jeangros C, Tarr PE. “We treat humans, not herds!”: A qualitative study of complementary and alternative medicine (CAM) providers’ individualized approaches to vaccination in Switzerland. Social Science & Medicine. 2019 Nov 1;240:112,556	Switzerland	2019	Our study aims at understanding CAM providers' roles in VH and asks the following questions: (1) how do CAM providers describe their perspectives and roles regarding vaccination?; (2) in what ways, if any, do CAM providers’ views and practices diverge from biomedical and public health vaccination discourses?; and (3) how do CAM providers and parents discuss vaccination during consultations?	Qualitative, 17 interviews (***7 anthroposophic providers***) and observed during vaccination consultations (*N* = 18 observations with 5 providers) employed individualized approaches to vaccination	Alternative/Complementary Anthroposophic health providers

**Table 5:** Coding tree from thematic analysis of qualitative data that explores factors influencing vaccine decision making in anthroposophic communities

Categories	Theme
*Acceptance of some vaccines*	*Broad spectrum of vaccine decisions*
*Vaccine delay*	
*Individualized vaccine schedules*	
*Vaccine decline*	
*Concern over side-effects*	*Consistent narrative about problems with vaccines*
*Toxicity*	
*Distrust in those recommending vaccines*	
*Individual vaccine schedules*	*Agency and independent thinking*
*Stigmatized from outside of the community – strengthens community*	*Stigma and social cohesion*
*Vaccine questioning / being part of the community (stigmatized when not questioned)*	

### Broad spectrum of vaccine decisions

All studies describe a ***broad spectrum of vaccine decisions*** *[theme 1]* [23, 37, 40–42]. There are those who *delay vaccines,* and the primary reason is to not overburden a young child’s body [23]. There are those who are positive towards some vaccines [23]; for instance, the tetanus vaccine appears to be accepted in several studies, yet often with a *delay* [41]. There are also some people who *vaccinate according to individual nee*d; for example, if they live on a farm, they vaccinate all their children against tetanus or if they do not think they can care for their child at home they vaccinate against MMR [41]. Similarly, several studies mention that parents vaccinate because there is an absence of disease and they would vaccinate their children in a setting with a high risk of the disease, e.g. when travelling abroad [23, 41].

Lastly, all six articles mentioned some groups in the anthroposophic community who decline vaccines altogether. Primarily this is due to the belief that childhood diseases are natural, natural immunity is better than vaccines, and because of concerns about vaccine content [23, 38, 40, 41]. Some anthroposophic health providers share the belief that diseases and fever are good for children and that they protect against allergies [38, 42]. The articles describe very little information about how vaccine decisions are made, apart from mentioning the important role and influence of peers and the community [41]. Sobo describes how some participants express authority and clear reasoning in their vaccine decision-making by drawing on scientific evidence [41]. However, the quality of that evidence is questioned, but not examined in detail.

### Consistent narrative about problems with vaccines

The articles describe a *consistent narrative about problems with vaccines [theme 2]*, particularly *concerns over side effects* of vaccines [23, 41–43]. Some papers expressed participants’ concerns with long-term side effects that may affect the brain due to aluminium found in some vaccines [42] and links to autoimmune diseases [41, 42]. Some anthroposophic health providers share the concerns about long-term effects on brain health and also add that vaccinated children are more likely to develop allergies and asthma [42]. Parental *concerns about toxicity* and how they interfere with long-term health were mentioned [40, 41]. A common argument against vaccine use expressed by both parents and anthroposophic healthcare providers was that vaccines interfere with children’s natural and necessary disease progression [23, 41–43]. Distrust in those producing vaccines for the sole purpose of profit was expressed in several papers [23, 41, 42].

### Agency and independent thinking

All studies consistently highlight that for both the anthroposophic community and anthroposophic healthcare providers, independent thinking and agency is an essential part of vaccine and health decision-making *[theme 3].* Moreover, the development of an *individualized vaccination schedule* is highly important [23, 38, 40–42]. Parents see themselves as making a well-informed choice and they take pride in their choice. Sobo summarises this idea by stating that *Alternative choices were taken to symbolize one’s capacity for independent thinking* [41]. Similarly, anthroposophic healthcare providers highlight the importance of a tailored approach that allows for individual freedom of choice [38, 42]. Individualized vaccination schedules were strongly advocated in all papers [23, 38, 40–42], as put by Sobo *“going along with the herd is not in keeping with the Waldorf ethos”* [41]. Due to ample scepticism towards vaccines and parents wanting to select the diseases to vaccinate against, some papers advocated for the importance of offering single rather than combined vaccines [38, 41].

### Stigma and social cohesion

Participants in the studies describe two types of *stigma associated with their vaccine beliefs [theme 4]*. On the one hand, they describe *stigma regarding their vaccine choices from the community outside of their anthroposophic community* as well as from mainstream health professionals [23, 40, 41]. Participants in the studies describe a sense of security they gain by sticking together in their communities: "*I have chosen to live here* [an anthroposophic community] *to be surrounded by people who have similar beliefs so that I do not have to stand up for myself all the time."* [23] In several papers, participants describe pride, hard work, and courage in that they are not simply following mainstream ideas. To summarize this in the words of a participant: "committing to Waldorf education *“takes courage” because it is so unconventional …It shows that the parents are individual thinkers... it takes a lot of work to go against the grain of society”* [40, 41]. Paradoxically, Sobo describes a *stigma to conform from within the anthroposophic community*, particularly in a Waldorf school setting. Parents describe that they actually do have different thoughts about vaccines than the community but fear to share those because they would threaten the social cohesion of the Waldorf identity. In Sobo’s words: “*Waldorfian identity make it harder and harder to contravene the norm without threatening one’s sense of group membership, or creating cognitive dissonance”* [39].

## Discussion

This systematic review showed that there have been a number of measles outbreaks associated with anthroposophic communities throughout Europe between the late 1990s and 2012 and one mumps outbreak. Vaccination catch-up was not a popular strategy in the anthroposophic community, but instead, the importance of school closure was highlighted. Outbreaks were not reported after 2012, it is unclear whether this is because there is a lack of research or no new outbreaks occured. This review further suggests that vaccination coverage is lower in anthroposophic communities compared to other communities, but evidence for this was somewhat weak and most focusing on MMR vaccines. The focus on MMR was arguably due to numerous measles outbreaks associated with anthroposophic communities. It would be important to understand specific vaccination coverage in more detail. For example, there would be value in understanding coverage for adolescent vaccines such as the human papillomavirus (HPV) vaccines and meningitis vaccines. Recent studies have highlighted the important effect the HPV vaccine on the reduction of cervical cancer – therefore understanding the views of parents from the anthroposophic community on the HPV vaccine would have important public health implications.

In terms of the qualitative findings, the review revealed a broad range of vaccination beliefs and highlighted the importance of individual choice in the vaccine decision process. Although parents consider themselves well-informed, it is unclear from the studies where they obtain their information, although some refer to the use of scientific information. Understanding knowledge and information sources in greater detail would be helpful to understand how certain rumours are maintained. The challenge of reliance on poor information sources to make a vaccine decision was particularly noted during the COVID-19 pandemic and arguably hindered COVID-19 vaccine uptake [44]. Some information, for example, the assumed link between low vaccination coverage and the development of allergies has been scientifically addressed and disproven [45]. Yet, the argument that a link persist was described in the literature. This arguably highlights that scientific results have not been effectively shared.

Although there are currently 27 articles that have investigated the relationship between vaccines and anthroposophy, it remains somewhat nebulous why anthroposophy as a religion or belief system is often considered as an anti-vax movement by popular media. Anthroposophical medicine does not reject vaccines, nor does it reject modern medicine. This was clearly stated at the inception of anthroposophical medicine and it has been a clear statement by Gotheanum. In 1925, Dr Rudolf Steiner and Dr Ita Wegman clearly stated *“It is not a matter of being in opposition to the school of medicine that is working with the accepted scientific methods of the present time. We fully acknowledge its principles. …we therefore feel compelled to work for a*n extension of clinical medicine*, based on these wider insights into the nature of the world and the human being” *[46]*.*

Sobo’s article described in this review was the only paper that engaged with the anthroposophic movement, particularly the Waldorf school context that arguably cultivated vaccine hesitancy [41]. The notion of building social cohesion through vaccine beliefs and decisions is an interesting and understudied concept. Understanding this further could perhaps help inform strategies to empower individuals to make their own decisions. For example, health providers engage with the question of how to address pressure to not vaccinate during their consultations. It would be interesting to further understand how stigma surrounding vaccine choices has changed in the context of COVID-19 vaccine decision-making. Furthermore, if the school and community context is a strong factor influencing vaccine decision-making, public health communication efforts should prioritize collaboration with the broader community rather than only health professionals working in that community. Given the low trust in public health authorities described in several studies in this review, this process will require a sensitive approach to avoid further alienation of the group.

The anthroposophic community prides itself on being different, communal, and supportive as opposed to following principles of consumerism and individualism. However, none of the studies, except briefly by Sobo, mentioned vaccines as a means for social action and to protect the vulnerable [41]. Distrust and the feeling of exclusion may be one of the reasons for this but perhaps it is a limited understanding of how vaccines actually work.

Lastly, the stigma this group experiences highlights a problem that requires careful attention. This could also be an important finding for other so-called vaccine hesitant groups [47]. One could argue that the more the anthroposophic group gets labelled as anti-vaxxers in public media or identified as vaccine hesitant by Public Health Agencies, the stronger their views become. Vaccine decision making, therefore, is no longer about individual and public health but rather linked to group identities. The research on the anthroposophic community has been somewhat limited in recent years. It would be important to continue to monitor vaccine sentiments in the anthroposophic community, particularly in view of the introduction of the COVID-19 vaccine and hesitancy linked with political sentiments [48, 49] and in view of emerging vaccines.

### Limitation

There are some limitations to this systematic review. The review only includes peer-reviewed articles; this means that there have probably been other disease outbreaks linked to anthroposophic communities, which were only described in the grey literature. Moreover, some of the studies purely described the outbreaks rather than conducted an analysis; therefore, it is difficult to analyse in depth what actually happened. Regarding the thematic analysis of the qualitative studies, there are limits to conducting such an analysis of results of existing studies, since we could not base our analysis on the full data set.

## Conclusion

This systematic review showed that there have been several measles outbreaks linked to anthroposophic communities in Europe. Although studies on vaccination coverage in anthroposophic communities are limited, it appears that coverage is lower than in the general population. Monitoring outbreak numbers and vaccination coverage could be important. Popular beliefs about the anthroposophic communities’ vaccination beliefs are challenged in this review. As the evidence shows the communities are not categorically against vaccines. Moreover, there are a myriad of factors that influence vaccine decision-making of parents belonging to an anthroposophic community. The importance of experiencing childhood illnesses and concerns over long-term side effects were mentioned. Moreover, parents want to be able to individually select vaccines for their children. They consider themselves actively engaged in vaccine decision-making and well-informed. Stigma regarding vaccine choices was mentioned repeatedly mostly by people outside of the anthroposophic community but also by people within the community. This review calls for a better understanding of vaccine choices and beliefs for vaccines beyond MMR, in particular HPV vaccines. The review also highlights a potentially important research gap, which constitutes understanding not only a belief system but the role that stigma may play in making decisions about vaccines.

### Supplementary Information

**Additional file 1.**

## Data Availability

The datasets used and/or analysed during the current study available from the corresponding author on reasonable request.

## Footnotes

1 Note that some papers discuss several outbreaks in one paper.
