# Supplementary material for: Understanding and explaining the link between anthroposophy and vaccine hesitancy: a systematic review
Source: BMC Public Health. 2023 Nov 13;23:2238. doi: 10.1186/s12889-023-17081-w (PMC10644591; doi:10.1186/s12889-023-17081-w)
Supplement: Supplementary file 1 — Additional file 1. [file 12889_2023_17081_MOESM1_ESM.docx]

**Appendix**

1. Medline

| Interface: Ovid MEDLINE(R) and Epub Ahead of Print, In-Process & Other Non-Indexed Citations and Daily  Date of Search: September 5:th, 2022  Number of hits: 87  Comment: In Ovid, two or more words are automatically searched as phrases; i.e. no quotation marks are needed | Field labels   - exp/ = exploded MeSH term - / = non exploded MeSH term - .ti,ab,kf. = title, abstract and author keywords - adjx = within x words, regardless of order - * = truncation of word for alternate endings |
| --- | --- |
| Database(s): **Ovid MEDLINE(R) and Epub Ahead of Print, In-Process, In-Data-Review & Other Non-Indexed Citations and Daily**1946 to September 02, 2022 Search Strategy:   \| **#** \| **Searches** \| **Results** \| \| --- \| --- \| --- \| \| 1 \| (anthroposoph* or steiner or waldorf*).ti,ab,kf. \| 1537 \| \| 2 \| exp Anthroposophy/ \| 241 \| \| 3 \| 1 or 2 \| 1604 \| \| 4 \| exp Vaccines/ \| 264705 \| \| 5 \| (vaccin* or unvaccin* or un vaccin* or nonvaccin* or non vaccin* or immunizat* or nonimmunizat* or immune* or nonimmune* or non immune* or unimmune* or un immune* or jab*).ti,ab,kf. \| 1120263 \| \| 6 \| 4 or 5 \| 1165980 \| \| 7 \| 3 and 6 \| 87 \| | |

2. Web of Science Core Collection

| Interface: Clarivate Analytics  Editions = A&HCI , ESCI , SCI-EXPANDED , SSCI  Date of Search: September 5:th, 2022  Number of hits: 104 | Field labels   - TS/Topic = title, abstract, author keywords and Keywords Plus - NEAR/x = within x words, regardless of order - * = truncation of word for alternate endings   Note: the *Exact search*-function was used for all the searches |
| --- | --- |
| \| Number \| Search \| Result \| \| --- \| --- \| --- \| \| 1 \| TS=(anthroposoph* or steiner or waldorf*) \| 8270 \| \| 2 \| TS=(vaccin* or unvaccin* or "un vaccin*" or nonvaccin* or "non vaccin*" or immunizat* or nonimmunizat* or immune* or nonimmune* or "non immune*" or unimmune* or "un immune*" or jab*) \| 1221125 \| \| 3 \| #1 AND #2 \| 104 \| | |

3. Psycinfo

| Interface: EBSCO  Date of Search: September 5:th, 2022  Number of hits: 9 | Field labels   - DE = subject heading - TI = title - AB = abstract - KW = author keywords - Nx = within x words, regardless of order - * = truncation of word for alternate endings |
| --- | --- |
| \| Number \| Search \| Result \| \| --- \| --- \| --- \| \| 1 \| TI anthroposoph* or steiner or waldorf* \| 103 \| \| 2 \| AB anthroposoph* or steiner or waldorf* \| 746 \| \| 3 \| KW anthroposoph* or steiner or waldorf* \| 98 \| \| 4 \| S1 OR S2 OR S3 \| 769 \| \| 5 \| DE "Vaccination Attitudes" \| 319 \| \| 6 \| TI vaccin* or unvaccin* or "un vaccin*" or nonvaccin* or "non vaccin*" or immunizat* or nonimmunizat* or immune* or nonimmune* or "non immune*" or unimmune* or "un immune*" or jab* \| 9807 \| \| 7 \| AB vaccin* or unvaccin* or "un vaccin*" or nonvaccin* or "non vaccin*" or immunizat* or nonimmunizat* or immune* or nonimmune* or "non immune*" or unimmune* or "un immune*" or jab* \| 35595 \| \| 8 \| KW vaccin* or unvaccin* or "un vaccin*" or nonvaccin* or "non vaccin*" or immunizat* or nonimmunizat* or immune* or nonimmune* or "non immune*" or unimmune* or "un immune*" or jab* \| 12717 \| \| 9 \| S5 OR S6 OR S7 OR S8 \| 37530 \| \| 10 \| S4 AND S9 \| 9 \| | |

4. Cinahl

| Interface: Ebsco  Date of Search: September 5:th, 2022  Number of hits: 38 | Field labels   - MH+ = exploded Cinahl Heading - MH = non exploded Cinahl Heading - TI = title - AB = abstract - Nx = within x words, regardless of order - * = truncation of word for alternate endings |
| --- | --- |
| \| Number \| Search \| Result \| \| --- \| --- \| --- \| \| 1 \| (MH "Anthroposophy") \| 509 \| \| 2 \| TI anthroposoph* or steiner or waldorf* \| 397 \| \| 3 \| AB anthroposoph* or steiner or waldorf* \| 860 \| \| 4 \| S1 OR S2 OR S3 \| 1298 \| \| 5 \| (MH "Vaccines+") \| 54417 \| \| 6 \| TI vaccin* or unvaccin* or "un vaccin*" or nonvaccin* or "non vaccin*" or immunizat* or nonimmunizat* or immune* or nonimmune* or "non immune*" or unimmune* or "un immune*" or jab* \| 64202 \| \| 7 \| AB vaccin* or unvaccin* or "un vaccin*" or nonvaccin* or "non vaccin*" or immunizat* or nonimmunizat* or immune* or nonimmune* or "non immune*" or unimmune* or "un immune*" or jab* \| 110360 \| \| 8 \| S5 OR S6 OR S7 \| 154636 \| \| 9 \| S4 AND S8 \| 38 \| | |
